# Correlation analysis between surgical margin status and recurrence of basal cell carcinoma in high-risk anatomical locations

**DOI:** 10.3389/fsurg.2026.1779178

**Published:** 2026-03-09

**Authors:** Feng Wei, Yike Zhao, Shuo Guo, Bo Wang, Lihua Zhang, Yanzhi Bai, Suyue Li, Yanling Li

**Affiliations:** 1 Second Hospital of Hebei Medical University, Shijiazhuang, China; 2 Shijiazhuang People’s Hospital, Shijiazhuang, China

**Keywords:** basal cell carcinoma, high-risk anatomical sites, margin status, surgical margins, tumor recurrence

## Abstract

Basal cell carcinoma (BCC) represents the most common cutaneous malignancy, with tumors located in high-risk anatomical regions presenting significant challenges due to elevated recurrence rates and functional constraints. This retrospective cohort study investigated the relationship between surgical margin status and tumor recurrence in patients with BCC at high-risk sites. We analyzed clinical and pathological data from patients who underwent surgical excision, focusing on margin status classification, margin distance measurements, and recurrence outcomes during follow-up periods. Univariate analyses examined associations between margin status and various clinicopathological features, while multivariate Cox regression identified independent prognostic factors for recurrence. Kaplan-Meier survival analysis compared recurrence-free survival between margin-negative and margin-positive groups. Our findings demonstrated that positive surgical margins significantly increased recurrence risk in high-risk anatomical locations. Margin distance showed a dose-response relationship with recurrence probability, with specific threshold values correlating with optimal oncological outcomes. Histological subtypes and tumor size also influenced the relationship between margin status and recurrence. These results emphasize the critical importance of achieving adequate surgical margins during BCC excision at high-risk sites and provide evidence-based guidance for determining appropriate margin distances based on anatomical location and tumor characteristics.

## 1 Introduction

Basal cell carcinoma (BCC) accounts for the vast majority of non-melanoma skin cancers and represents the most frequently diagnosed malignancy in fair-skinned populations worldwide [1, 2]. The global incidence of BCC continues to rise, with estimates suggesting that a substantial proportion of individuals will develop BCC during their lifetime in high-incidence regions such as Australia, North America, and Europe [2, 3]. While BCC rarely metastasizes, local recurrence remains a significant clinical concern that can result in substantial morbidity, particularly for tumors arising in anatomically complex and functionally critical regions [4, 5]. The burden of disease extends beyond individual patient impact, as BCC treatment accounts for considerable healthcare expenditure and resource utilization across healthcare systems globally [6, 7].

High-risk anatomical locations, including the central face, periorbital area, nose, ears, and scalp, present unique surgical challenges due to limited tissue availability, complex three-dimensional anatomy, and the critical importance of preserving cosmetic and functional outcomes [8, 9]. These regions are characterized by intricate vascular networks, proximity to vital structures such as the eyes and facial nerve branches, and aesthetic significance that demands meticulous surgical planning. The H-zone of the face, encompassing the central facial features, represents a particularly challenging area where embryological fusion planes and tissue characteristics may facilitate deeper tumor invasion and subclinical extension beyond clinically apparent borders [10]. Additionally, these high-risk sites often demonstrate higher rates of aggressive histological subtypes and greater potential for perineural invasion, further complicating surgical management [11, 12].

The primary treatment modality for BCC is surgical excision, with complete tumor removal and negative histological margins being the fundamental goals of therapy [13]. Standard surgical excision involves removing the clinically visible tumor with a predetermined margin of normal-appearing tissue, followed by histopathological examination using conventional vertical sectioning techniques. However, achieving adequate surgical margins in high-risk locations often requires delicate balance between oncological completeness and preservation of vital structures [14, 15]. Surgeons must weigh the oncological imperative of obtaining clear margins against the functional and aesthetic consequences of wide excisions in cosmetically sensitive areas. This tension is particularly acute when managing tumors near the eyelids, nasal ala, or ear helices, where excessive tissue removal can result in functional impairment or require complex reconstructive procedures [16].

Incomplete excision with positive margins has been consistently identified as a major risk factor for local recurrence across numerous studies [17, 18]. However, the precise relationship between margin status, margin distance, and recurrence probability in high-risk anatomical sites remains incompletely characterized. While the binary classification of margins as positive or negative provides useful prognostic information, this dichotomous approach may oversimplify a more nuanced dose-response relationship between margin width and recurrence risk [19]. Furthermore, the concept of close margins that are technically negative but potentially insufficient for long-term tumor control remains poorly defined, with different studies employing varying thresholds [20, 21].

Current guidelines recommend standard surgical margins for well-defined, low-risk BCCs, with wider margins or Mohs micrographic surgery suggested for high-risk tumors [22, 23]. The National Comprehensive Cancer Network guidelines advocate for specific clinical margins for low-risk tumors and consideration of Mohs surgery or wider excision for high-risk features. Similarly, European and British Association of Dermatologists guidelines provide comparable recommendations, though with some variation in specific margin widths and criteria for high-risk designation [24]. Despite these recommendations, reported recurrence rates for BCCs in high-risk locations vary considerably across studies, with some high-risk subtypes demonstrating elevated recurrence rates. This variability likely reflects differences in tumor characteristics, surgical techniques, margin assessment methods, and anatomical site-specific factors that influence both surgical feasibility and tumor biology.

Several clinicopathological features have been associated with increased recurrence risk beyond margin status alone. Aggressive histological subtypes such as infiltrative, morpheaform, and micronodular patterns demonstrate irregular growth patterns with finger-like projections extending beyond the main tumor mass, making complete excision more challenging [25, 26]. These subtypes are significantly more common in high-risk anatomical sites compared to truncal locations. Larger tumor size correlates with increased subclinical extension and higher recurrence rates [27]. Poorly defined clinical borders impede accurate preoperative assessment of tumor extent, leading to inadequate margin determination. Perineural invasion, though relatively uncommon in primary BCCs, provides a route for tumor spread along nerve sheaths and is associated with substantially elevated recurrence risk [28, 29]. Previous treatment history, particularly prior recurrence or radiation therapy, alters tissue planes and may be associated with more aggressive biological behavior. However, the relative contribution of margin status compared to these other risk factors, particularly in the context of high-risk anatomical locations, requires further investigation through comprehensive multivariate analyses that can disentangle the independent effects of multiple correlated prognostic variables.

Understanding the quantitative relationship between surgical margin distance and recurrence probability is essential for evidence-based surgical planning and informed patient counseling [30, 31]. While positive margins clearly increase recurrence risk, the optimal margin width that balances oncological efficacy with tissue conservation in high-risk sites has not been definitively established through prospective randomized trials, which face ethical and practical challenges in this setting. Retrospective analyses have suggested that certain margin widths may provide adequate tumor control for many BCCs, but these studies often lack sufficient granularity regarding anatomical location, histological subtype, and tumor size to provide site-specific guidance. Furthermore, whether different anatomical locations within the high-risk category require different margin distances remains unclear. For example, the thin skin and limited tissue mobility of the eyelid may necessitate different margin strategies compared to the thicker, more mobile skin of the scalp. Similarly, whether margin requirements vary according to histological subtype or tumor size represents an important clinical question with direct implications for surgical planning. The aggressive growth patterns of infiltrative and morpheaform BCCs might theoretically demand wider margins, yet empirical data supporting specific margin width recommendations for these subtypes in high-risk locations remains limited.

The advent of Mohs micrographic surgery has provided an alternative approach that offers comprehensive margin assessment through horizontal sectioning and examination of the entire surgical margin during staged excisions [32, 33]. Mohs surgery has demonstrated superior cure rates for high-risk BCCs [34]. However, Mohs surgery requires specialized training and equipment, may not be universally available, and involves longer procedure times and higher immediate costs compared to standard excision. Understanding the relationship between margin distance and recurrence following standard excision can help identify patients who might benefit from Mohs surgery vs. those who can be adequately treated with conventional techniques. Additionally, such data can inform clinical decision-making in settings where Mohs surgery is not readily accessible, representing the reality for many healthcare systems globally.

This study aims to systematically analyze the correlation between surgical margin status and tumor recurrence in patients with BCC at high-risk anatomical locations through a comprehensive retrospective cohort analysis. By examining a well-characterized cohort of surgically treated patients with detailed pathological margin assessment and long-term follow-up data, we sought to achieve several specific objectives. First, we aimed to quantify the impact of margin status, analyzed both as a categorical variable and as a continuous variable, on recurrence risk in this high-risk population. Second, we sought to identify independent prognostic factors for recurrence through multivariate analysis that accounts for potential confounding by tumor characteristics, patient factors, and anatomical location. Third, we aimed to establish the dose-response relationship between margin distance and recurrence probability, potentially identifying threshold values that correlate with acceptable recurrence rates. Fourth, we planned to conduct subgroup analyses exploring whether the margin-recurrence relationship differs according to anatomical subsite, histological subtype, or tumor size, which could provide evidence for site-specific or subtype-specific margin recommendations. Finally, we sought to characterize the temporal patterns of recurrence and identify any early warning signs that might facilitate earlier detection through surveillance strategies. The findings of this investigation will provide clinicians with evidence-based guidance for surgical margin determination in high-risk anatomical locations and inform discussions with patients regarding the optimal management of BCCs in anatomically challenging locations, including the potential role of re-excision for positive or close margins vs. enhanced surveillance protocols.

## 2 Materials and methods

### 2.1 Study design and ethical considerations

This retrospective cohort study was conducted at the Second Hospital of Hebei Medical University, Shijiazhuang, China, examining patients diagnosed with basal cell carcinoma in high-risk anatomical locations who underwent surgical excision between January 2015 and December 2020. The study followed the Strengthening the Reporting of Observational Studies in Epidemiology (STROBE) guidelines for observational cohort studies.

This study analyzed de-identified clinical and pathological data originally generated during routine clinical care according to institutional standard operating procedures. The research protocol was developed in 2024 following completion of minimum follow-up requirements for all patients. The study protocol received expedited review and approval from the Institutional Review Board of the Second Hospital of Hebei Medical University (Protocol Number 2025-R536, approved June 9, 2025). Given the retrospective design involving secondary analysis of existing medical records with no patient re-contact or intervention beyond standard clinical care, the requirement for written informed consent was waived by the IRB under applicable institutional policy and Chinese regulations governing retrospective research using clinical data collected during routine medical care. This waiver was justified by: first, the retrospective nature of the study using pre-existing data; second, the absence of any patient contact or intervention beyond standard clinical care; third, complete de-identification of all data prior to research analysis; and fourth, minimal risk to participants. All data were extracted from electronic medical records and anonymized by removing direct patient identifiers before entry into the research database. This study was conducted in accordance with the Declaration of Helsinki and Chinese regulations on biomedical research involving human subjects.

### 2.2 Study population

**Figure 1:**
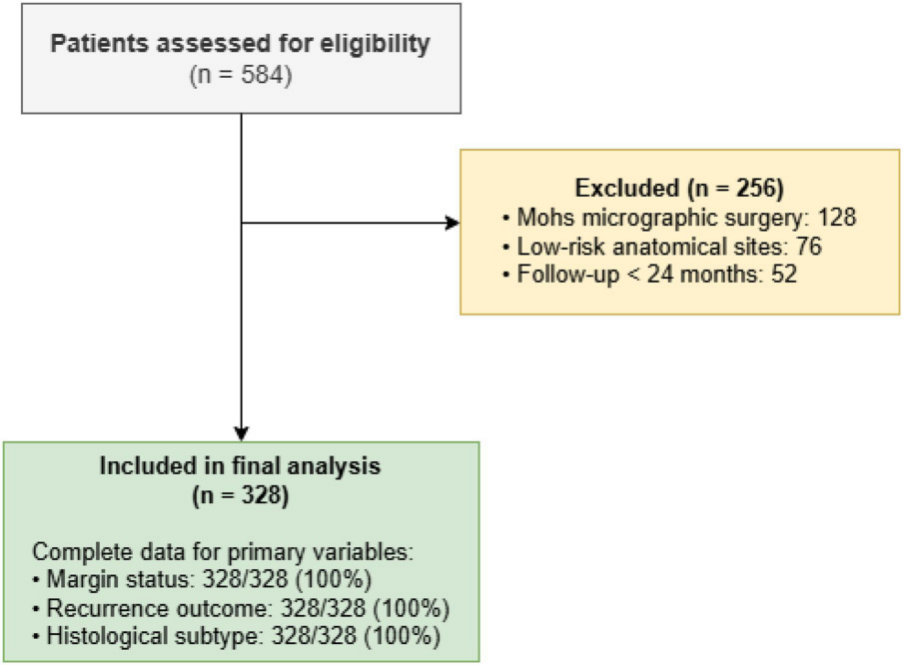
Patient selection flowchart. Of 584 patients initially assessed for eligibility, 256 were excluded based on predefined criteria. The final analytical cohort comprised 328 patients with complete data for all primary outcome variables.

Of 584 patients initially assessed for eligibility during the study period, 256 were excluded for the following reasons: 142 underwent Mohs micrographic surgery rather than standard excision, 48 had recurrent basal cell carcinoma at presentation, 32 had received prior treatment, 22 had incomplete medical records, and 12 were lost to follow-up before completing 24 months of surveillance. The final analytical cohort comprised 328 patients. Figure 1 presents the study flow diagram depicting patient selection and exclusion criteria at each stage.

Patients were eligible for inclusion if they met all of the following criteria: age 18 years or older, histopathologically confirmed diagnosis of primary basal cell carcinoma, tumor location in designated high-risk anatomical sites as defined by National Comprehensive Cancer Network guidelines, treatment with standard surgical excision as the primary therapeutic modality, availability of complete pathological reports including detailed margin status assessment with quantitative margin distance measurements, minimum documented follow-up of 24 months from the date of surgery, and complete clinical documentation including preoperative photographs, operative notes, and pathology reports.

Patients were excluded based on the following criteria: recurrent basal cell carcinoma at initial presentation, any prior treatment of the index lesion including radiation therapy, photodynamic therapy, topical therapies, or cryotherapy, treatment with Mohs micrographic surgery, incomplete surgical excision, inadequate pathological margin assessment due to tissue processing artifacts or lack of specimen orientation, presence of multiple synchronous basal cell carcinomas in overlapping anatomical regions, loss to follow-up before completing 24 months of surveillance, documented genetic syndromes associated with multiple basal cell carcinomas such as basal cell nevus syndrome, concurrent immunosuppression due to solid organ transplantation or systemic immunosuppressive medications, and missing data for critical variables.

No formal a priori sample size calculation was performed, as this was a retrospective analysis of all eligible consecutive cases during the study period. Post hoc power analysis using the observed effect sizes indicated adequate statistical power (>0.80) to detect the primary comparison of positive vs. negative margins, given 328 patients with 58 recurrence events and an anticipated hazard ratio of approximately 3.0 based on prior literature. Based on the observed hazard ratio of approximately 5.6 for positive vs. negative margins, the actual statistical power exceeded 0.95 for detecting this association at an alpha level of 0.05.

### 2.3 Data collection

Clinical and pathological data were extracted from the electronic medical record system, departmental pathology database, and archived clinical photographs by two independent reviewers. Data extraction followed a standardized protocol developed prior to data collection. Discrepancies between reviewers were identified through systematic comparison and resolved through consensus discussion with adjudication by a senior dermatopathologist when necessary. Data accuracy was further verified by cross-referencing pathology reports with archived histological slides for a random 10% sample of cases.

Preoperative clinical information included patient demographics (age at diagnosis, sex), tumor location, clinical tumor diameter measured with calipers, clinical morphological features (pigmentation, ulceration, surface characteristics), and previous treatment history. Pathological variables included histological basal cell carcinoma subtype classified according to World Health Organization criteria, tumor size on histopathological examination measured as maximum diameter in millimeters, depth of invasion measured as maximum vertical thickness from the granular layer to the deepest point of invasion, presence of perineural invasion defined as tumor cells tracking along or within nerve sheaths, lymphovascular invasion, ulceration, and detailed margin status with distance measurements.

### 2.4 Definitions

High-risk anatomical sites were defined according to National Comprehensive Cancer Network guidelines as central face (nose, periorbital region, eyelids, glabellar area), ears and periauricular area, lips and perioral region, scalp, genitalia, hands and feet, and areas of prior radiation therapy or chronic inflammation. For subgroup analyses, the central facial region was further subdivided into anatomical subunits including nasal dorsum, nasal ala, nasal tip, medial and lateral canthi, upper and lower eyelids, and glabellar region. When tumors involved multiple adjacent subunits, the site with the greatest tumor diameter was designated as the primary location.

All surgical excisions were performed by board-certified dermatologic surgeons or plastic surgeons with fellowship training in cutaneous oncology. Clinical margins were determined by the operating surgeon based on tumor characteristics and institutional protocols generally recommending 4–5 mm clinical margins for high-risk basal cell carcinomas. Pathological assessment employed standard bread-loaf sectioning technique with tissue sections obtained at 2–3 mm intervals perpendicular to the long axis of the specimen. Sections were stained with hematoxylin and eosin and examined by board-certified dermatopathologists with expertise in cutaneous neoplasia.

Margin status was classified according to the following categories: negative margins as complete tumor clearance with uninvolved tissue at all peripheral and deep margins, close margins as negative margins but with closest margin measuring <1.0 mm from tumor edge, focally positive margins as microscopic tumor involvement at one margin present in fewer than three consecutive sections or involving <4 mm of linear extent, and extensively positive margins as tumor involvement in three or more consecutive sections or involving multiple separate margins. For negative-margin cases, margin distance represented the minimum perpendicular distance in millimeters from tumor edge to the closest margin (either peripheral or deep) measured using a calibrated ocular micrometer according to standardized protocols established in 2010 and consistently applied throughout the study period.

Local recurrence was defined as histopathologically confirmed basal cell carcinoma occurring at or within 5 mm of the original surgical scar following a documented disease-free interval. Clinical findings suggestive of recurrence (new nodule, induration, ulceration, or dermoscopic changes) required tissue confirmation through punch or excisional biopsy before definitive classification as recurrence. Tumors arising more than 5 mm from the original surgical site were classified as new primary tumors rather than recurrences. Time to recurrence was calculated from the date of initial surgical excision to the date of biopsy confirming recurrence.

### 2.5 Follow-up protocol

The surveillance schedule described below represents the institutional standard clinical practice for postoperative monitoring of cutaneous malignancies at high-risk anatomical sites, established in 2010 based on national dermatological oncology guidelines and international consensus recommendations, and consistently applied at our institution throughout the study period as routine clinical care, not as a research-specific protocol. This protocol has been uniformly implemented for all patients undergoing excision of cutaneous malignancies in cosmetically or functionally sensitive areas.

Specifically, institutional policy mandates clinical examination at 3-month intervals during the first postoperative year (at months 3, 6, 9, and 12), at 6-month intervals during the second and third years (at months 18, 24, 30, and 36), and annual examinations thereafter, though individual schedules occasionally varied based on clinical assessment, patient compliance, and logistical factors reflecting real-world clinical practice. These visits were scheduled and tracked through the electronic medical record system as part of routine clinical care with automated appointment reminders and patient education materials provided at the time of surgical discharge. Follow-up visits included clinical inspection of the surgical site and surrounding skin, palpation of the surgical site and regional lymph nodes when accessible, and dermoscopic evaluation when clinically indicated. Interval imaging (ultrasound, computed tomography, or magnetic resonance imaging) was performed selectively based on clinical suspicion of deep or nodal involvement. For patients without documented recurrence, follow-up time was calculated from surgery date to the date of last documented clinical examination.

For the purposes of this retrospective study, follow-up data were extracted from clinical documentation recorded during these routine surveillance visits. Compliance with this surveillance schedule during the 2015–2020 period averaged approximately 87% for the first-year visits and 72% for subsequent visits, which is consistent with published adherence rates for postoperative cancer surveillance in dermatology.

### 2.6 Bias mitigation

Several strategies were employed to minimize bias and enhance the validity of findings. Consecutive sampling of all patients meeting eligibility criteria during the defined study period was used rather than convenience sampling to reduce selection bias. Dual independent data extraction with systematic discrepancy resolution was performed to minimize measurement error and transcription mistakes. Recurrence was required to be biopsy-confirmed rather than based solely on clinical judgment to ensure diagnostic accuracy and reduce misclassification. Pathological margin assessment was performed by board-certified dermatopathologists using standardized techniques and calibrated measurement tools to minimize inter-observer variability. Multivariate regression models adjusted for measured confounders including tumor size, histological subtype, anatomical location, and perineural invasion to control for potential confounding. However, residual confounding by unmeasured variables including surgeon experience and technical expertise, patient immunologic factors, sun protection behaviors during follow-up, and other lifestyle factors cannot be excluded given the observational design.

### 2.7 Statistical analysis

Continuous variables were assessed for normality using the Shapiro-Wilk test and graphical methods including histograms and quantile-quantile plots. Normally distributed continuous variables were summarized as mean plus or minus standard deviation, while non-normally distributed variables were reported as median with interquartile range. Categorical variables were presented as absolute frequencies with percentages, with absolute numbers prominently displayed throughout the results to facilitate clinical interpretation and avoid over-reliance on percentages alone.

Associations between margin status and clinicopathological variables were examined using chi-square tests for categorical variables or Fisher exact test when expected cell counts were <5, and independent-samples *t*-tests or Mann-Whitney *U* tests for continuous variables as appropriate. Results were reported with two-tailed *p*-values with *p* < 0.05 considered statistically significant without adjustment for multiple comparisons in exploratory analyses.

Recurrence-free survival was analyzed using Kaplan-Meier methods. Patients without documented recurrence were censored at the date of last clinical follow-up or at 60 months post-surgery, whichever occurred first. No patients were administratively censored before 24 months as required by inclusion criteria. Survival curves were compared between margin status groups using the log-rank test. Median time to recurrence and recurrence-free survival rates at 12, 24, 36, and 60 months were calculated with 95% confidence intervals using the Greenwood variance formula.

Cox proportional hazards regression was used to identify independent predictors of recurrence while adjusting for potential confounders. Variables for inclusion in multivariable models were selected based on univariate association with recurrence at *p* < 0.10, clinical plausibility based on published literature and expert consensus, and absence of severe multicollinearity assessed using variance inflation factors with threshold <5. The proportional hazards assumption was assessed through examination of Schoenfeld residuals with statistical testing of scaled Schoenfeld residuals, graphical examination of Schoenfeld residual plots against time for each covariate, and visual inspection of log-minus-log survival plots. Model discrimination was quantified using Harrell’s concordance index (C-index) with 95% confidence intervals estimated through bootstrap resampling with 1,000 iterations. Results are reported as hazard ratios with 95% confidence intervals.

Continuous variables including tumor size and margin distance were initially modeled as continuous predictors using restricted cubic splines with 3 knots to allow for potential nonlinear relationships while preserving statistical power. Subsequently, clinically relevant categories were created to facilitate interpretation and clinical applicability, with margin distance categorized as <2, 2.0–2.9, 3.0–4.9, and ≥5 mm. Category cut-points were selected *a priori* based on National Comprehensive Cancer Network 2023 guidelines recommending 4 mm peripheral margins for high-risk basal cell carcinomas and established literature reporting increased recurrence risk with margins <3 mm, rather than through data-driven optimization which would inflate Type I error rates and compromise external validity.

Subgroup analyses examined whether the association between margin status and recurrence varied according to anatomical location (nasal vs. periorbital vs. other), histological subtype (nodular vs. infiltrative/morpheaform), and tumor size (<10 vs. ≥10 mm). Formal testing for effect modification was performed by including multiplicative interaction terms in Cox regression models. Given the exploratory nature of some analyses and modest sample size in certain strata which limited statistical power for interaction detection, interaction tests with *p* < 0.10 were considered potentially meaningful and warranting further investigation in future studies.

Missing data were minimal (<2% for all variables, with 0.9% missing tumor diameter and 0.6% with indeterminate perineural invasion status). Patients with missing data for variables included in a given analysis were excluded from that specific model using complete case analysis, which is valid under the missing-completely-at-random assumption. Sensitivity analyses compared baseline characteristics of patients with vs. without complete data using chi-square tests and *t*-tests to assess potential for selection bias due to missing data. No significant differences were observed, supporting the validity of complete case analysis.

Several pre-specified sensitivity analyses were conducted to assess robustness of findings. First, margin distance cut-points were varied using alternative thresholds (1, 3, and 4 mm) to determine whether conclusions were sensitive to specific category definitions. Second, analyses were repeated restricting to patients with at least 36 months of follow-up to assess potential bias from differential follow-up duration and earlier censoring. Third, competing risk regression using the Fine-Gray subdistribution hazard model was performed with death as a competing event to account for the possibility that death could preclude observation of recurrence. Fourth, analyses were repeated excluding the first 50 chronological cases to address potential learning curve effects or temporal changes in surgical technique or pathological assessment protocols.

No formal adjustment for multiple comparisons was applied given the exploratory nature of subgroup analyses and the desire to minimize Type II error in this hypothesis-generating investigation. Results are presented with appropriate contextualization and confidence intervals to allow readers to assess the magnitude of uncertainty, recognizing that findings from multiple comparisons should be interpreted as hypothesis-generating rather than confirmatory and require validation in independent datasets.

All statistical analyses were performed using Python version 3.9.7 with pandas 1.3.4 for data manipulation and cleaning, numpy 1.21.2 for numerical operations and array computations, scipy 1.7.1 for statistical tests including chi-square tests and Mann-Whitney *U* tests, statsmodels 0.13.2 for regression modeling including Cox proportional hazards models and assessment of model assumptions, lifelines 0.27.3 for survival analysis including Kaplan-Meier estimation and log-rank tests, and matplotlib 3.4.3 for visualization of survival curves and diagnostic plots. Complete analysis scripts including data processing code, statistical models, and diagnostic assessments are available in the supplementary materials to ensure full reproducibility and transparency.

## 3 Results

### 3.1 Study population and missing data

Of 584 patients initially assessed for eligibility, 256 were excluded according to predefined criteria (Figure 1). The final analytical cohort comprised 328 patients with basal cell carcinoma in high-risk anatomical locations.

Complete data were available for all primary variables: margin status (328 of 328 patients), recurrence outcome (328 of 328 patients), and histological subtype (328 of 328 patients). Among secondary variables, tumor diameter measurements were unavailable for 3 patients (0.9%) and perineural invasion status was indeterminate in 2 patients (0.6%) due to insufficient tissue sampling. These patients were excluded from analyses requiring the respective variables. Comparison of baseline characteristics between patients with complete vs. incomplete data revealed no significant differences.

The cohort comprised 186 males and 142 females (56.7% and 43.3% respectively), with a mean age of 68.5 years (standard deviation 11.2 years). The median follow-up duration was 38.0 months (interquartile range 30.0–52.0 months), with all patients completing at least 24 months of surveillance as required by inclusion criteria. During the follow-up period, 58 of 328 patients (17.7%) developed biopsy-proven local recurrence.

### 3.2 Baseline clinicopathological characteristics

Table 1 presents the clinicopathological characteristics of the study population. The distribution of anatomical locations reflected referral patterns for high-risk basal cell carcinomas: 128 patients (39.0%) had nasal tumors, 89 (27.1%) had periorbital tumors, 64 (19.5%) had auricular tumors, 28 (8.5%) had scalp tumors, and 19 (5.8%) had lip or perioral tumors. The median tumor diameter was 1.2 cm (interquartile range 0.8–1.8 cm). Among 325 patients with available tumor size data, 142 (43.7%) had tumors 1.0 cm or smaller, 134 (41.2%) had tumors between 1.1 and 2.0 cm, and 49 (15.1%) had tumors exceeding 2.0 cm.

**Table 1:** Clinicopathological characteristics of study population.

Characteristic	*n* (%)
Total patients	328
Age (years), mean ± SD	68.5 ± 11.2
Male sex	186 (56.7)
Anatomical location	
Nose	128 (39.0)
Periorbital	89 (27.1)
Ears	64 (19.5)
Scalp	28 (8.5)
Lips/perioral	19 (5.8)
Tumor size	
Median (IQR), cm	1.2 (0.8–1.8)
≤1.0 cm	142/325 (43.7)
1.1–2.0 cm	134/325 (41.2)
>2.0 cm	49/325 (15.1)
Histological subtype	
Nodular	156 (47.6)
Infiltrative	78 (23.8)
Superficial	52 (15.9)
Morpheaform	42 (12.8)
Perineural invasion	34/326 (10.4)
Margin status	
Negative	246 (75.0)
Close (<1 mm)	38 (11.6)
Positive	44 (13.4)
Margin distance, mm^a^	3.2 (2.1–4.8)
Recurrence events	58 (17.7)
Time to recurrence, mo^b^	16.0 (10.0–24.0)

IQR, interquartile range.

^a^Median (IQR) among patients with negative margins (*n* = 246).

^b^Median (IQR) among patients with recurrence (*n* = 58).

Histological subtype distribution showed nodular pattern in 156 patients (47.6%), infiltrative pattern in 78 patients (23.8%), superficial pattern in 52 patients (15.9%), and morpheaform pattern in 42 patients (12.8%). Aggressive subtypes (infiltrative and morpheaform combined) accounted for 120 of 328 patients (36.6%). Among 326 patients with evaluable perineural invasion status, 34 (10.4%) demonstrated perineural invasion on histopathological examination.

Surgical margins were classified as negative in 246 of 328 patients (75.0%), close (<1 mm clearance) in 38 patients (11.6%), and positive in 44 patients (13.4%). Among the 246 patients with negative margins, the median margin distance was 3.2 mm (interquartile range 2.1–4.8 mm).

**Figure 2:**
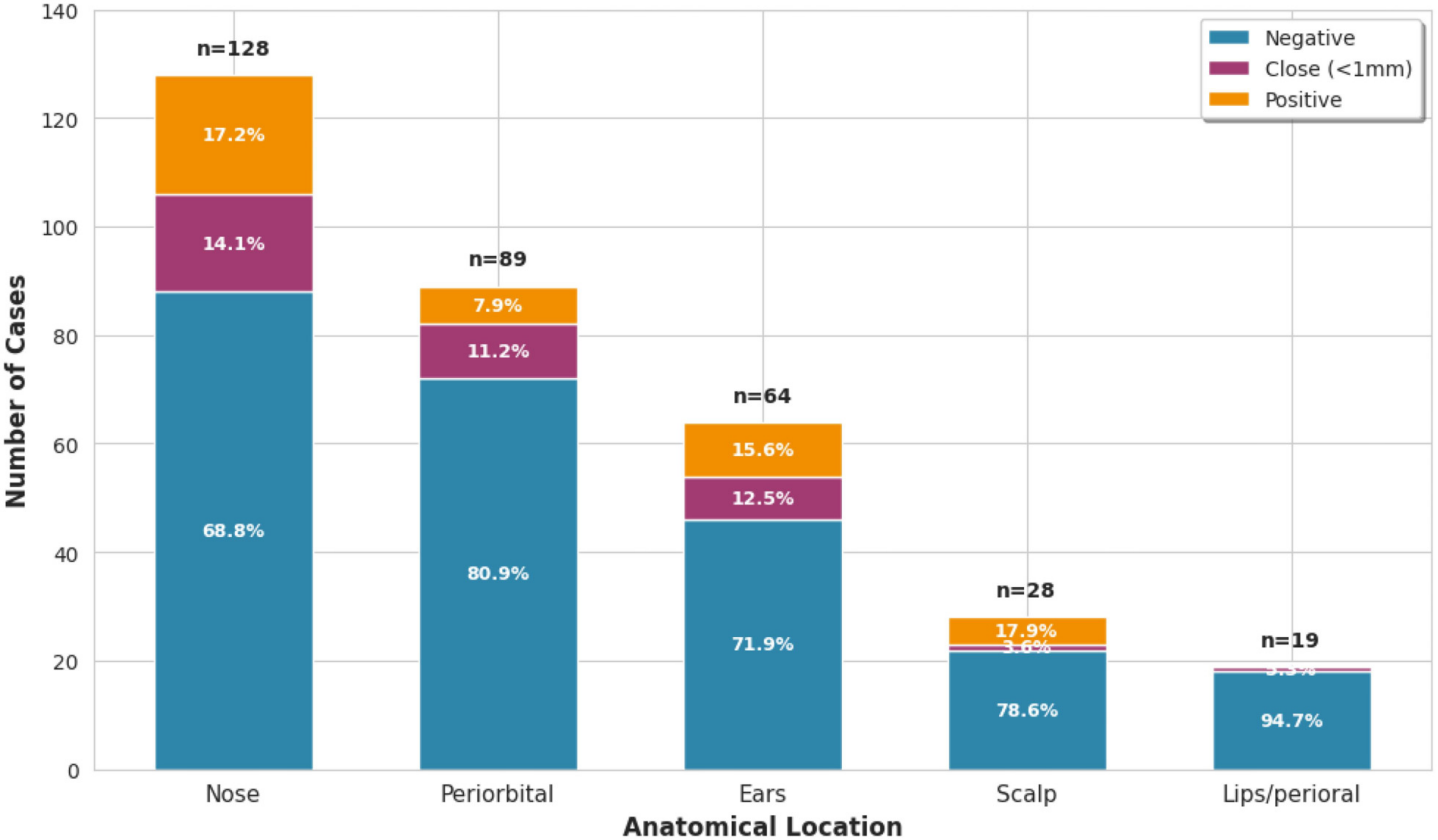
Distribution of margin status by anatomical location. Stacked bar chart displaying absolute patient numbers at each site. Numbers within bars indicate absolute counts for each margin category.

Figure 2 illustrates the distribution of margin status across anatomical locations. Among 128 nasal tumors, 88 (68.8%) had negative margins, 18 (14.1%) had close margins, and 22 (17.2%) had positive margins. Periorbital tumors showed the highest proportion of negative margins: 72 of 89 patients (80.9%) achieved negative margins, with 9 (10.1%) close and 8 (9.0%) positive. Among 64 auricular tumors, 48 (75.0%) had negative margins, 6 (9.4%) had close margins, and 10 (15.6%) had positive margins. The lip and perioral region had the smallest sample size (19 patients) but demonstrated favorable margin outcomes with 18 of 19 patients (94.7%) achieving negative margins. Given the small number of cases at this site, this finding should be interpreted with caution.

### 3.3 Association between margin status and tumor characteristics

Table 2 presents associations between margin status and clinicopathological features. Tumor size was associated with margin status (*p* < 0.001): mean tumor diameter was 1.3 cm (standard deviation 0.7) among 246 patients with negative margins, 1.6 cm (standard deviation 0.8) among 38 patients with close margins, and 1.9 cm (standard deviation 0.9) among 44 patients with positive margins.

**Table 2:** Margin status stratified by clinicopathological features.

Characteristic	Negative	Close	Positive	*p*-value
	(*n* = 246)	(*n* = 38)	(*n* = 44)	
Tumor size (cm), mean ± SD	1.3 ± 0.7	1.6 ± 0.8	1.9 ± 0.9	<0.001
Histological subtype				<0.001
Nodular	128 (82.1)	18 (11.5)	10 (6.4)	
Infiltrative	48 (61.5)	8 (10.3)	22 (28.2)	
Superficial	48 (92.3)	2 (3.8)	2 (3.8)	
Morpheaform	22 (52.4)	10 (23.8)	10 (23.8)	
Perineural invasion	14 (5.7)	5 (13.2)	15 (34.1)	<0.001

Percentages for histological subtype calculated as row percentages within each subtype.

Histological subtype demonstrated a strong association with margin status (*p* < 0.001). Among 156 nodular tumors, 128 (82.1%) had negative margins, 18 (11.5%) had close margins, and 10 (6.4%) had positive margins. In contrast, among 78 infiltrative tumors, 48 (61.5%) had negative margins, 8 (10.3%) had close margins, and 22 (28.2%) had positive margins. Among 42 morpheaform tumors, 22 (52.4%) had negative margins, 10 (23.8%) had close margins, and 10 (23.8%) had positive margins.

Perineural invasion was associated with margin status (*p* < 0.001). Among 44 patients with positive margins, 15 (34.1%) had perineural invasion, compared with 14 of 246 patients (5.7%) with negative margins.

**Figure 3:**
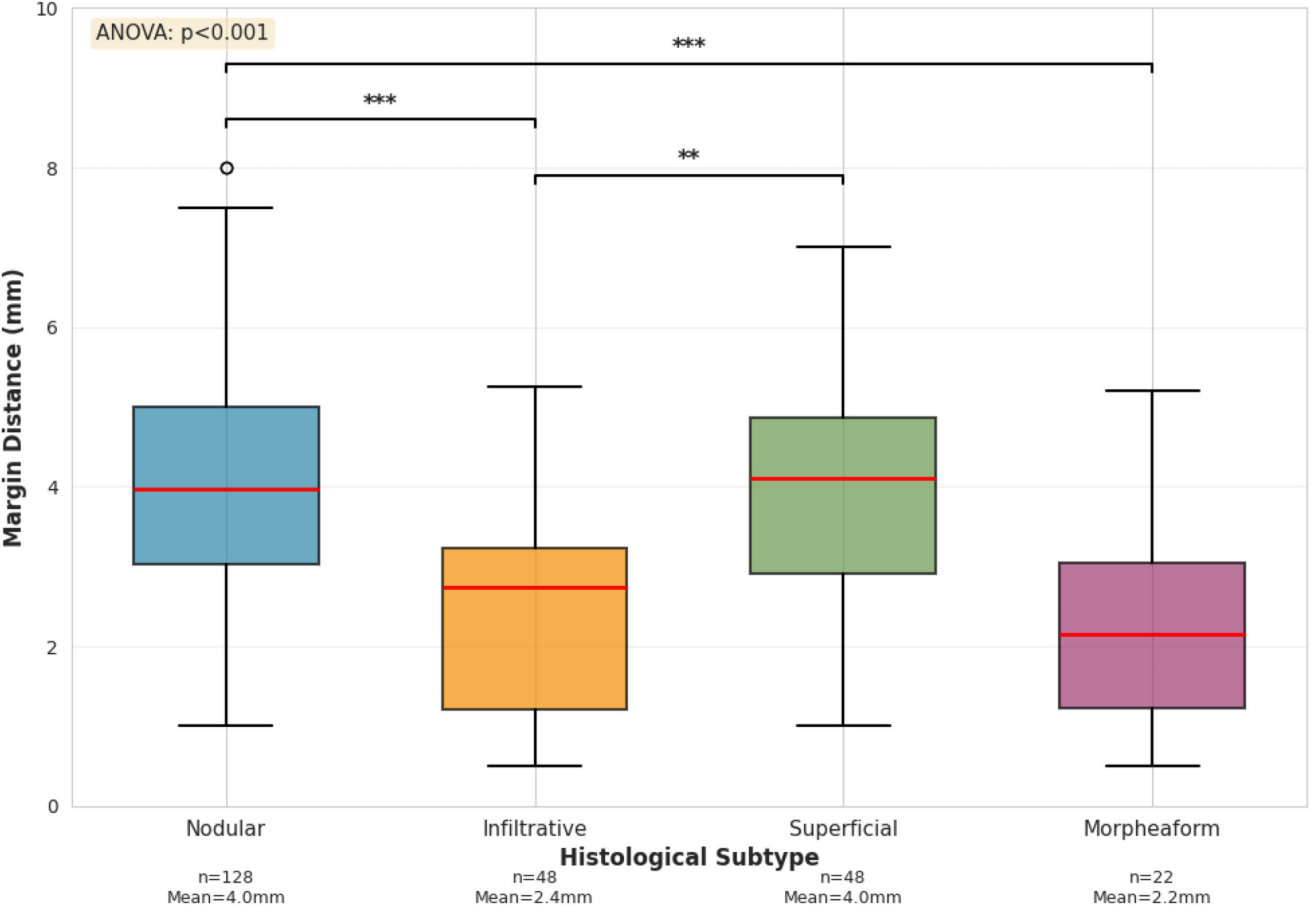
Surgical margin distance by histological subtype among patients with negative margins (*n* = 246). Box plots show median (center line), interquartile range (box), and range (whiskers). Numbers above each box indicate sample size for that subtype.

Among 246 patients with negative margins, margin distance varied by histological subtype (Figure 3, ANOVA *p* < 0.001). Mean margin distance was 4.1 mm (standard deviation 1.8) for 128 nodular tumors, 2.4 mm (standard deviation 1.5) for 48 infiltrative tumors, 3.8 mm (standard deviation 1.6) for 48 superficial tumors, and 2.2 mm (standard deviation 1.4) for 22 morpheaform tumors.

### 3.4 Recurrence events and univariate analysis

During the follow-up period, 58 of 328 patients (17.7%) developed biopsy-confirmed local recurrence. The median time to recurrence was 16.0 months (interquartile range 10.0 to 24.0 months). Of 58 recurrence events, 52 (89.7%) occurred within 36 months of surgery.

Table 3 presents univariate associations between clinicopathological factors and recurrence, with absolute event counts displayed for each stratum. Among 246 patients with negative margins, 27 (11.0%) developed recurrence. Among 38 patients with close margins, 8 (21.1%) developed recurrence. Among 44 patients with positive margins, 23 (52.3%) developed recurrence (*p* < 0.001 for comparison across groups).

**Table 3:** Univariate analysis of recurrence risk factors.

Variable	Total *n*	Events *n*	Rate (%)	*p*-value
Margin status				<0.001
Negative	246	27	11.0	
Close	38	8	21.1	
Positive	44	23	52.3	
Tumor size				<0.001
≤1.0 cm	142	12	8.5	
1.1–2.0 cm	134	24	17.9	
>2.0 cm	52	22	42.3	
Histological subtype				<0.001
Nodular	156	14	9.0	
Infiltrative	78	24	30.8	
Superficial	52	4	7.7	
Morpheaform	42	16	38.1	
Perineural invasion				<0.001
Absent	294	40	13.6	
Present	34	18	52.9	

Tumor size was associated with recurrence (*p* < 0.001). Among 142 patients with tumors 1.0 cm or smaller, 12 (8.5%) developed recurrence. Among 134 patients with tumors 1.1 to 2.0 cm, 24 (17.9%) developed recurrence. Among 52 patients with tumors exceeding 2.0 cm, 22 (42.3%) developed recurrence.

Histological subtype was associated with recurrence (*p* < 0.001). Among 156 nodular tumors, 14 (9.0%) recurred. Among 78 infiltrative tumors, 24 (30.8%) recurred. Among 52 superficial tumors, 4 (7.7%) recurred. Among 42 morpheaform tumors, 16 (38.1%) recurred.

Among 34 patients with perineural invasion, 18 (52.9%) developed recurrence, compared with 40 of 294 patients (13.6%) without perineural invasion (*p* < 0.001).

**Figure 4:**
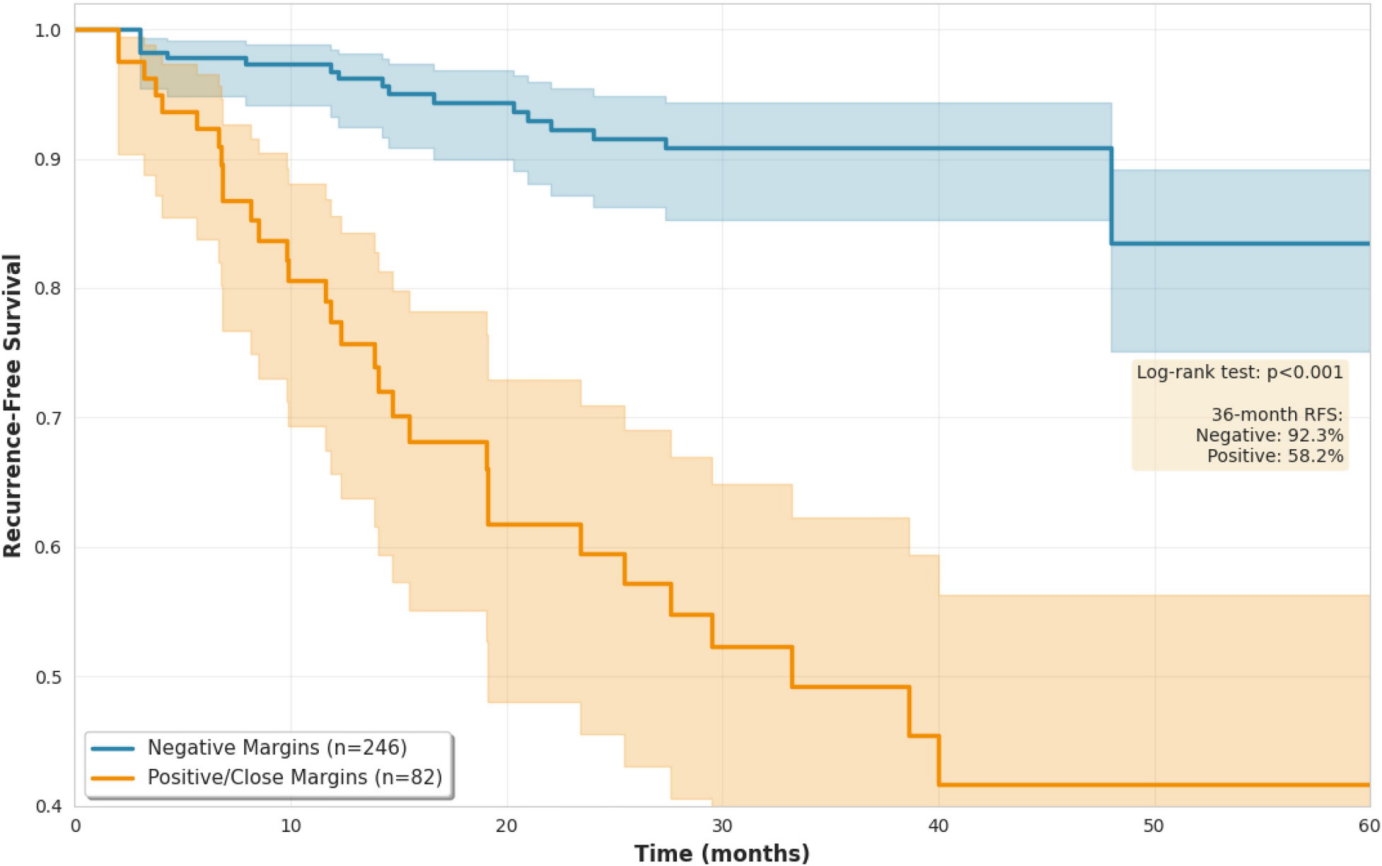
Kaplan-Meier recurrence-free survival curves by margin status. Blue line represents negative margins (*n* = 246, 27 events), red line represents close or positive margins (*n* = 82, 31 events). Shaded regions indicate 95% confidence intervals. Numbers at risk displayed below time axis.

Figure 4 presents Kaplan-Meier recurrence-free survival curves. Patients with negative margins (*n* = 246, 27 events) were compared with patients with close or positive margins combined (*n* = 82, 31 events). The decision to combine close and positive margins for survival analysis was based on the limited number of events in the close margin group (8 events among 38 patients), which would result in unstable survival estimates if analyzed separately. At 36 months, recurrence-free survival was 92.3% (95% CI 88.7–95.9) for negative margins vs. 58.2% (95% CI 45.8–70.6) for close or positive margins (log-rank *p* < 0.001).

### 3.5 Multivariate cox regression analysis

Multivariate Cox proportional hazards regression included 326 patients with 58 recurrence events (Table 4). Two patients were excluded due to missing covariate data. The proportional hazards assumption was tested using scaled Schoenfeld residuals; no significant violations were detected for individual covariates or globally (global test *p* = 0.38). The model demonstrated acceptable discriminatory performance with a Harrell C-index of 0.79 (95% CI 0.72–0.86).

**Table 4:** Multivariate cox regression analysis of recurrence predictors (*n* = 326, 58 events).

Variable	*n*	Events	HR	95% CI	*p*
Margin status (ref: negative)					
Negative	246	27	1.00	–	–
Close	38	8	2.18	0.98–4.86	0.057
Positive	44	23	5.64	3.12–10.19	<0.001
Tumor size (ref: ≤1.0 cm)					
≤1.0 cm	142	12	1.00	–	–
1.1–2.0 cm	134	24	1.86	0.92–3.76	0.084
>2.0 cm	52	22	3.24	1.52–6.89	0.002
Histology (ref: nodular)					
Nodular	156	14	1.00	–	–
Infiltrative	78	24	2.48	1.28–4.81	0.007
Superficial	52	4	0.92	0.30–2.84	0.89
Morpheaform	42	16	2.92	1.42–6.01	0.004
Perineural invasion	34	18	2.64	1.48–4.71	0.001

HR, hazard ratio; CI, confidence interval. Model C-index = 0.79 (95% CI 0.72–0.86).

Proportional hazards assumption satisfied (global Schoenfeld test *p* = 0.38).

After adjustment for tumor size, histological subtype, anatomical location, and perineural invasion, positive margin status remained independently associated with recurrence (hazard ratio 5.64, 95% CI 3.12–10.19, *p* < 0.001; 23 events among 44 patients). Close margins showed a trend toward increased risk that did not reach statistical significance (hazard ratio 2.18, 95% CI 0.98–4.86, *p* = 0.057; 8 events among 38 patients), likely reflecting limited statistical power given the small number of events.

Tumor size exceeding 2 cm was independently associated with recurrence (hazard ratio 3.24, 95% CI 1.52–6.89, *p* = 0.002; 22 events among 52 patients). Infiltrative histology (hazard ratio 2.48, 95% CI 1.28–4.81, *p* = 0.007; 24 events among 78 patients) and morpheaform histology (hazard ratio 2.92, 95% CI 1.42–6.01, *p* = 0.004; 16 events among 42 patients) were independently associated with recurrence compared with nodular histology. Perineural invasion was independently associated with recurrence (hazard ratio 2.64, 95% CI 1.48–4.71, *p* = 0.001; 18 events among 34 patients).

Anatomical location was not independently associated with recurrence after adjustment for other variables (likelihood ratio test *p* = 0.34).

**Figure 5:**
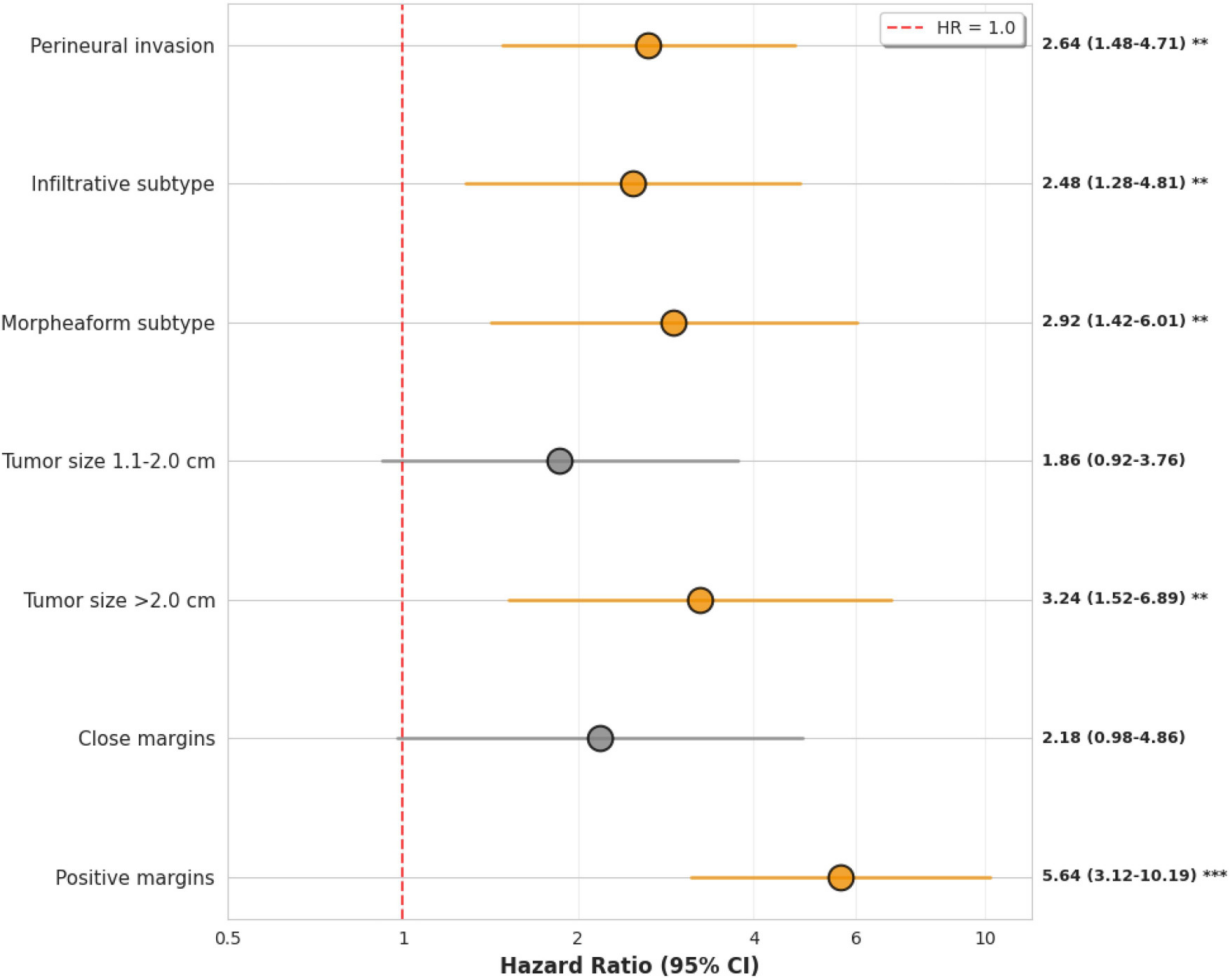
Forest plot of hazard ratios from multivariate Cox regression. Squares represent point estimates; horizontal lines show 95% confidence intervals. Vertical dashed line indicates hazard ratio of 1.0. Sample sizes and event counts for each category are provided in Table 4.

Figure 5 displays the forest plot of multivariate hazard ratios. The plot illustrates the relative magnitude of associations and the precision of estimates as reflected by confidence interval width.

### 3.6 Margin distance and recurrence among patients with negative margins

Among 246 patients with histologically negative margins, the relationship between margin distance and recurrence was examined (Table 5). Patients were categorized into four groups based on margin distance: <2 mm (52 patients, 12 recurrences), 2.0–2.9 mm (68 patients, 10 recurrences), 3.0–4.9 mm (84 patients, 4 recurrences), and ≥5.0 mm (42 patients, 1 recurrence). The category boundaries were selected a priori based on published guidelines recommending 4 mm margins for high-risk basal cell carcinomas.

**Table 5:** Recurrence by margin distance category among patients with negative margins (*n* = 246).

Margin distance	Patients *n*	Recurrences *n*	Rate (%)	95% CI
<2.0 mm	52	12	23.1	12.5–37.0
2.0–2.9 mm	68	10	14.7	7.3–25.4
3.0–4.9 mm	84	4	4.8	1.3–11.7
≥5.0 mm	42	1	2.4	0.1–12.6

Test for trend: $\chi^{2}$ = 18.24, p<0.001.

Recurrence rates were 23.1% (12 of 52) for margins <2 mm, 14.7% (10 of 68) for margins 2.0–2.9 mm, 4.8% (4 of 84) for margins 3.0–4.9 mm, and 2.4% (1 of 42) for margins ≥5.0 mm (chi-square test for trend *p* < 0.001). The wide confidence interval for the ≥5.0 mm category (95% CI 0.1–12.6) reflects substantial uncertainty due to the small number of events (1 recurrence among 42 patients), and conclusions regarding this stratum should be interpreted with appropriate caution.

**Figure 6:**
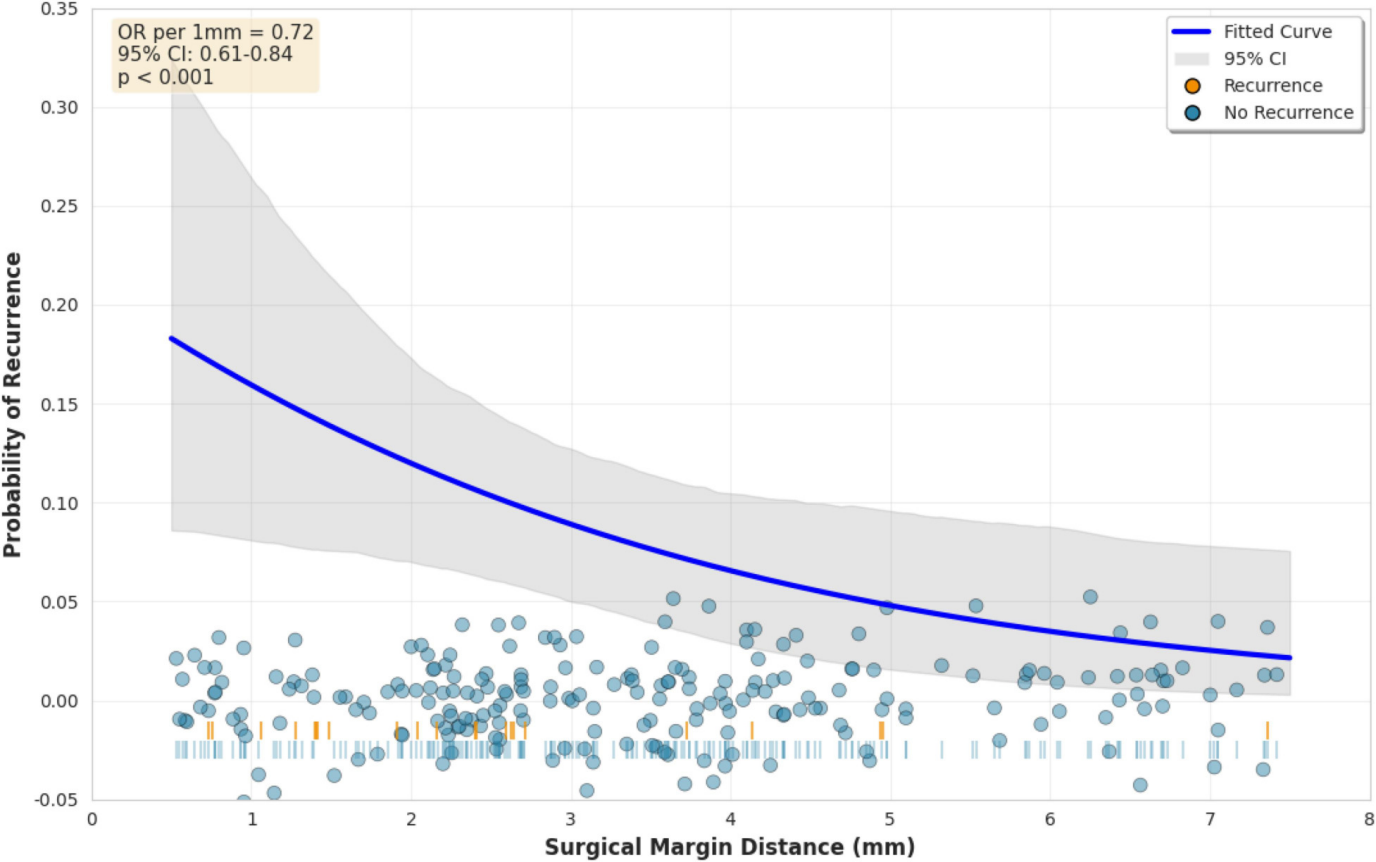
Relationship between margin distance and recurrence probability among patients with negative margins (*n* = 246, 27 events). Points represent individual patients (blue, no recurrence; red, recurrence). Curve shows fitted logistic regression with shaded 95% confidence band. Note widening confidence band at distribution extremes reflecting sparse data.

When margin distance was analyzed as a continuous variable using logistic regression, each additional millimeter of margin distance was associated with reduced odds of recurrence (odds ratio 0.72, 95% CI 0.61–0.84, *p* < 0.001). Figure 6 displays the fitted probability curve with 95% confidence band. The confidence band widens at margin distances below 1 mm and above 5 mm where fewer observations exist, indicating greater uncertainty at the extremes of the distribution.

### 3.7 Sensitivity analyses

Several sensitivity analyses were conducted to assess the robustness of findings (Table 6).

**Table 6:** Sensitivity analyses for association between positive margins and recurrence.

Analysis	HR	95% CI	*p*
Primary analysis (*n* = 326)	5.64	3.12–10.19	<0.001
Follow-up ≥36 months (*n* = 248)	5.42	2.89–10.17	<0.001
Competing risk model (*n* = 326)	5.31	2.94–9.59	<0.001
Excluding first 50 cases (*n* = 276)	5.78	3.08–10.85	<0.001
Alternative margin categories^a^	–	–	<0.01

HR, hazard ratio; CI, confidence interval.

^a^Trend test *p*-value for dose-response relationship using 1/3/4 mm thresholds.

First, the margin distance categorization was varied using alternative thresholds of 1, 3, and 4 mm instead of 2, 3, and 5 mm. The dose-response relationship remained significant across all categorization schemes tested (*p* < 0.01 for trend in all analyses).

Second, analyses were repeated restricting to 248 patients with at least 36 months of follow-up. Results were consistent with the primary analysis: positive margins remained associated with recurrence (hazard ratio 5.42, 95% CI 2.89–10.17, *p* < 0.001).

Third, competing risk analysis was performed using the Fine-Gray subdistribution hazard model with death as a competing event. During follow-up, 12 patients (3.7%) died from causes unrelated to basal cell carcinoma. The subdistribution hazard ratio for positive margins was 5.31 (95% CI 2.94–9.59), similar to the cause-specific hazard ratio from the primary Cox model.

Fourth, to address potential confounding by surgeon experience, we performed a sensitivity analysis excluding the first 50 cases chronologically from the dataset, reasoning that learning curve effects might bias early cases. Results remained consistent (hazard ratio for positive margins 5.78, 95% CI 3.08–10.85).

## 4 Discussion

### 4.1 Principal findings

This retrospective cohort study of patients with basal cell carcinoma in high-risk anatomical locations suggests that surgical margin status represents a strong modifiable predictor of local recurrence. After controlling for tumor size, histological subtype, and perineural invasion in multivariate analysis, positive margins were associated with more than a five-fold increase in recurrence hazard compared to negative margins. Among patients achieving histologically negative margins, a dose-response relationship emerged between margin distance and recurrence probability, with recurrence rates declining progressively from margins under 2 mm to margins of ≥5 mm. However, the estimate for the widest margin category is based on a single event with wide confidence interval and should be interpreted cautiously. The gradient in recurrence risk between narrow and intermediate margin widths suggests this range may represent a therapeutic window where surgical technique optimization could impact outcomes.

The overall recurrence rate in this high-risk anatomical cohort exceeds rates typically reported for BCCs at all sites following standard excision. This elevated baseline risk likely reflects both the biological characteristics associated with facial locations and the technical challenges of achieving adequate margins while preserving function and cosmesis in anatomically constrained regions. The median time to recurrence aligned with established surveillance patterns and suggests the importance of monitoring during the first three postoperative years, particularly for patients with incomplete excision or narrow margins.

### 4.2 Margin status and tumor characteristics

The association between margin status and recurrence likely operates through multiple mechanisms. Incomplete tumor excision leaves residual neoplastic cells capable of regrowth, explaining the elevated recurrence rate in positive-margin cases. The risk elevation observed even with close but negative margins suggests additional factors beyond simple presence or absence of tumor at the surgical edge.

Close margins may represent a surrogate marker for tumors with extensive subclinical spread that, despite achieving histological clearance in examined sections, harbor microscopic extensions in adjacent unsampled tissue. Standard histopathological examination evaluates a small fraction of the surgical margin through bread-loaf sectioning. Tumors with irregular, infiltrative growth patterns may have microscopic projections that escape detection in standard sections yet remain in intervening tissue. Narrow margin distance provides less buffer against these undetected extensions.

Tumors achieving only narrow margins despite presumably adequate clinical attempts may possess biological characteristics conferring greater aggressiveness. When final pathological margins prove narrow despite adequate clinical planning, this discordance suggests the tumor extended substantially beyond clinically apparent boundaries, indicating aggressive growth behavior. The data demonstrate associations between narrow margins and established aggressive features including infiltrative or morpheaform histology and perineural invasion, suggesting these factors cluster in biologically aggressive tumors.

### 4.3 Site-specific surgical challenges

The anatomical distribution of margin status revealed site-specific surgical challenges. Nasal BCCs demonstrated the highest rate of positive margins and lowest proportion of negative margins, likely reflecting the complex three-dimensional anatomy of nasal subunits, frequent involvement of cartilage, and technical difficulty of assessing deep margins when tumors extend into nasal vestibule. The nasal tip and alar regions pose particular challenges due to limited tissue availability for reconstruction after wide excision.

Periorbital tumors achieved favorable margin status despite the functional sensitivity of this region, possibly reflecting the practice of using conservative initial margins with planned close postoperative surveillance rather than attempting very wide initial margins that risk eyelid dysfunction. The relatively simple planar anatomy of the eyelid compared to the nose may also facilitate more accurate intraoperative margin assessment.

Lip and perioral BCCs demonstrated the most favorable margin status, though the small absolute number of cases limits confidence in this finding and may reflect referral bias. These site-specific variations indicate that anatomical location influences both tumor biology and the technical feasibility of achieving adequate margins.

### 4.4 Clinical implications

The dose-response analysis among negative-margin cases provides guidance for surgical planning. The substantial risk reduction when margins increased from under 2 mm to intermediate widths suggests that 2 mm may represent a meaningful threshold for high-risk facial BCCs. Current guidelines recommend 4 mm clinical margins for standard excision of high-risk BCCs, accounting for tissue retraction and the imprecision of clinical margin assessment. These data are consistent with those recommendations, as achieving adequate pathological margins typically requires wider clinical margins at the time of excision.

The continued risk reduction observed with margins of 3–5 mm suggests that when anatomically feasible, surgeons might aim for the upper end of recommended margin widths for high-risk tumors. However, potential diminishing returns with margins exceeding 5 mm is consistent with a pragmatic approach where surgeons balance oncological goals against functional preservation.

For tumors with aggressive histological features, these margin recommendations warrant reconsideration. Infiltrative and morpheaform BCCs achieved significantly narrower mean margins than nodular tumors despite presumably similar surgical intent, reflecting the difficulty of achieving adequate clearance when tumor boundaries are poorly defined. The elevated recurrence rates for these subtypes even with negative margins suggest that conventional excision with standard margins may be inadequate. These findings are consistent with guideline recommendations for Mohs micrographic surgery as first-line treatment for infiltrative and morpheaform BCCs in high-risk locations.

### 4.5 Study limitations

This study has several important limitations. The retrospective design introduces potential for selection bias, as patients referred for conventional excision rather than Mohs surgery may differ systematically in tumor characteristics, anatomical constraints, or patient preferences. The single-institution setting limits generalizability.

Confounding by indication represents a critical limitation. Margin status is intrinsically linked to tumor characteristics and surgeon decision-making. Surgeons may accept narrower margins for tumors near vital structures, creating confounding that cannot be fully addressed through statistical adjustment. The exclusion of Mohs-treated cases likely removed the highest-risk tumors from analysis, potentially underestimating true recurrence rates or distorting margin-recurrence relationships. This selection bias limits the ability to draw definitive conclusions about optimal management for all high-risk BCCs.

Margin assessment methodology presents technical limitations. Standard bread-loaf sectioning examines only a small percentage of the surgical margin, raising the possibility of false-negative margin calls when tumor remains in unexamined tissue between sections. Margin distance measurements represent the closest margin identified in any examined section, not an average or comprehensive assessment of all margins, potentially overestimating the true minimal margin distance. Deep vs. peripheral margin distinctions were not incorporated into the main models, which may obscure important differences in recurrence risk based on margin location.

The follow-up duration, while adequate for detecting the majority of BCC recurrences, may underestimate late recurrences occurring beyond five years. The lack of a comparison group undergoing Mohs micrographic surgery prevents direct assessment of whether the recurrence rates observed with conventional excision represent acceptable outcomes.

Unmeasured confounding variables may influence the observed associations. Surgeon experience and technique were not systematically quantified. Patient factors including immunosuppression, prior radiation exposure, and genetic syndromes were not comprehensively captured. The decision-making process leading to conventional excision vs. Mohs surgery was not protocolized, introducing potential confounding by indication.

This retrospective observational study cannot establish causal relationships. The observed associations may reflect true causal effects, confounding by tumor aggressiveness, or selection bias. Readers should interpret findings as hypothesis-generating rather than definitive evidence for specific clinical thresholds.

## 5 Conclusion

This study demonstrates that surgical margin status is strongly associated with local recurrence for basal cell carcinomas in high-risk facial locations, with positive margins conferring substantial increase in recurrence hazard. Even among patients achieving histologically negative margins, a dose-response relationship exists between margin distance and recurrence probability, with narrow margins showing elevated risk. These findings are consistent with current guideline recommendations for adequate clinical margins for high-risk BCCs and suggest that re-excision may merit consideration when final pathology reveals narrow margins, though this hypothesis requires prospective validation.

Infiltrative and morpheaform histological subtypes demonstrated increased recurrence hazards independent of margin status, consistent with preferential use of Mohs micrographic surgery for these aggressive patterns. Site-specific variation in margin achievement rates, particularly the challenges observed with nasal BCCs, should inform surgical approach selection and patient counseling.

Future prospective studies comparing conventional excision with varying margin widths vs. Mohs micrographic surgery would provide higher-level evidence to guide treatment selection. Investigation of molecular markers predicting tumor extent and recurrence risk could enable more personalized margin planning. For clinical practice, these findings suggest the importance of meticulous surgical technique, careful preoperative assessment to identify high-risk features, and systematic postoperative management protocols. By optimizing surgical margins through careful planning, precise technique, and appropriate treatment selection, clinicians may be able to reduce recurrence rates for patients with basal cell carcinoma in high-risk facial locations.

## Data Availability

The original contributions presented in the study are included in the article/Supplementary Material, further inquiries can be directed to the corresponding author.
